# *Runx* Family Genes in a Cartilaginous Fish, the Elephant Shark (*Callorhinchus milii*)

**DOI:** 10.1371/journal.pone.0093816

**Published:** 2014-04-03

**Authors:** Giselle Sek Suan Nah, Zhi Wei Lim, Boon-Hui Tay, Motomi Osato, Byrappa Venkatesh

**Affiliations:** 1 Institute of Molecular and Cell Biology, Agency for Science, Technology and Research, Singapore, Singapore; 2 School of Biological Sciences, Nanyang Technological University, Singapore, Singapore; 3 Cancer Science Institute of Singapore, National University of Singapore, Singapore, Singapore; 4 Institute of Bioengineering and Nanotechnology, Agency for Science, Technology and Research, Singapore, Singapore; 5 Department of Pediatrics, Yong Loo Lin School of Medicine, National University of Singapore, Singapore, Singapore; University of Massachusetts Medical, United States of America

## Abstract

The *Runx* family genes encode transcription factors that play key roles in hematopoiesis, skeletogenesis and neurogenesis and are often implicated in diseases. We describe here the cloning and characterization of *Runx1*, *Runx2*, *Runx3* and *Runxb* genes in the elephant shark (*Callorhinchus milii*), a member of Chondrichthyes, the oldest living group of jawed vertebrates. Through the use of alternative promoters and/or alternative splicing, each of the elephant shark *Runx* genes expresses multiple isoforms similar to their orthologs in human and other bony vertebrates. The expression profiles of elephant shark *Runx* genes are similar to those of mammalian *Runx* genes. The syntenic blocks of genes at the elephant shark *Runx* gene loci are highly conserved in human, but represented by shorter conserved blocks in zebrafish indicating a higher degree of rearrangements in this teleost fish. Analysis of promoter regions revealed conservation of binding sites for transcription factors, including two tandem binding sites for Runx that are totally conserved in the distal promoter regions of elephant shark *Runx1-3*. Several conserved noncoding elements (CNEs), which are putative *cis*-regulatory elements, and miRNA binding sites were identified in the elephant shark and human *Runx* gene loci. Some of these CNEs and miRNA binding sites are absent in teleost fishes such as zebrafish and fugu. In summary, our analysis reveals that the genomic organization and expression profiles of *Runx* genes were already complex in the common ancestor of jawed vertebrates.

## Introduction

The Runt domain transcription factor, known as the polyomavirus enhancer-binding protein 2 (PEBP2) or core-binding factor (CBF) is a heterodimer of α and β subunits. In humans, the α-subunit comprises three proteins, RUNX1, RUNX2 and RUNX3 that contain an evolutionarily conserved 128 amino acid Runt domain responsible for DNA binding and heterodimerization with the β-subunit. The β-subunit includes a single protein, RUNX β (also known as PEBP2β or CBFβ) that does not contain a DNA-binding domain but allosterically enhances the DNA-binding activity of the α-subunit and regulates its turnover by protecting it from ubiquitin-proteasome-mediated degradation [1], [2].

RUNX1-3 are key transcriptional regulators involved in several major developmental pathways including hematopoiesis, neurogenesis and skeletogenesis. *RUNX1* is among the most frequently mutated genes in human leukemias [3], [4], [5]. In mice, *Runx1* is critical for the generation and maintenance of hematopoietic stem cells (HSC) [6], [7]. In addition, *Runx1* is involved in the development of skeletal muscle [8], neurons [9] and hair follicles [10]. On the other hand, *Runx2* is indispensable for bone development, as evidenced by *Runx2-/-* mice which lack ossified skeleton and therefore die from respiratory failure shortly after birth [11]. In humans, haploinsufficiency of *RUNX2* is associated with cleidocranial dysplasia (CCD), an autosomal dominant skeletal disorder [12]. *Runx3* is expressed in a wide range of tissues and has diverse biological functions. It plays roles in the regulation of epithelial homeostasis in the gastrointestinal tract [13], [14], T-cell development during thymopoiesis [15] and differentiation of immune cells including natural killer cells [16], dendritic cells [17] and B cells [18]. *Runx3* is also crucial for the differentiation of proprioceptive dorsal root ganglion (DRG) neurons [19] and chondrocyte maturation during skeletogenesis [20]. In human, *RUNX3* has been implicated in a multitude of cancers where it can function as a tumor suppressor or oncogene [21], [22].

Given the importance of *Runx* genes in major developmental pathways and human diseases, orthologs of *Runx* have been characterized in phylogenetically diverse organisms to facilitate better understanding of their origin and functions. *Runx* genes have undergone duplications independently in some invertebrate lineages (e.g., fruit fly, mosquito) and in the stem vertebrate lineage followed by acquisition of specialized functions such as hematopoiesis and eye development in *Drosophila* and bone development in vertebrates [23], [24]. Tetrapods contain three *Runx* genes that are orthologs of human *RUNX1-3*. Among teleost fishes, pufferfishes (fugu and *Tetraodon*) contain four Runx genes [25], [26], of which three are orthologs of mammalian *Runx1-3*. The fourth gene, called *frRunt*, is hypothesized to represent an ancestral vertebrate *Runx* gene that was subsequently lost in tetrapods [25]. In zebrafish, besides *Runx1* and *Runx3*, there are two copies of *Runx2* (*Runx2a* and *Runx2b*) which likely arose from the whole-genome duplication event in the teleost fish ancestor [27]. In contrast to multiple copies of α-subunit encoding *Runx* genes, *Runxβ* is present as a single copy in all metazoans analysed [24]. Studies into the co-evolution of Runxα and Runxβ subunits have reported similar evolutionary rates of these interacting proteins and evolutionary conservation of the structure of the Runxα-Runxβ-DNA complex, suggesting that these proteins have co-evolved to maintain their ability to interact and to coregulate the transcription of target genes [24].

Cartilaginous fishes (Chondrichthyes) are phylogenetically the oldest living group of jawed vertebrates (Gnathostomes) that diverged from bony vertebrates (Osteichthyes) approximately 450 million years (My) ago [28]. By virtue of their phylogenetic position, cartilaginous fishes are a useful reference for the study of the origin and evolution of jawed vertebrate genes and their regulation. Cartilaginous fishes are split into two groups: elasmobranchs comprising sharks, rays and skates; and holocephalans represented by chimaeras such as elephant shark (*Callorhinchus milii*). Coding sequences of *Runx* genes were previously cloned in an elasmobranch, the dogfish (*Scyliorhinus canicula*) [29]. Three *Runx* genes, orthologous to mammalian *Runx1-3* were found to be expressed in the developing cartilage, teeth and placoid scales suggesting that they may be involved in the ancient processes of vertebrate skeletogenesis in this cartilaginous fish [29]. To improve our understanding of the evolution, function and regulation of *Runx* genes, we have now cloned *Runx* genes from the elephant shark and analysed their genomic organization. The elephant shark has the smallest genome among cartilaginous fishes and has been proposed as a model cartilaginous fish genome [30], [31]. Recently the whole genome sequence of the elephant shark was completed [32]. Its comparison with human and other vertebrate genomes indicated that elephant shark is the slowest evolving known vertebrate genome [32]. Furthermore, human and elephant shark were found to share twice as many conserved noncoding elements (CNE), which are putative *cis*-regulatory elements, as human and teleosts fishes [33]. Thus, elephant shark is a valuable reference genome for understanding the evolution of gene families and *cis*-regulatory networks in jawed vertebrates.

In this study, we have characterized three members of *Runx* family genes in the elephant shark, as well as the gene encoding the β-subunit, *CmRunxb*, by cloning of cDNA and mining the elephant shark genome database. We have determined the tissue-specific mRNA expression of *CmRunx* and showed that these patterns reflect those in mammalian tissues, which may point to evolutionarily conserved gene functions and developmental pathways. Comparisons of noncoding sequences in the elephant shark and other jawed-vertebrate *Runx* loci were able to identify CNEs that are conserved over 450 million years of vertebrate evolution and are likely to be *cis*-regulatory elements.

## Results and Discussion

### Cloning and characterization of elephant shark α-subunit Runx family members

**Figure 1:**
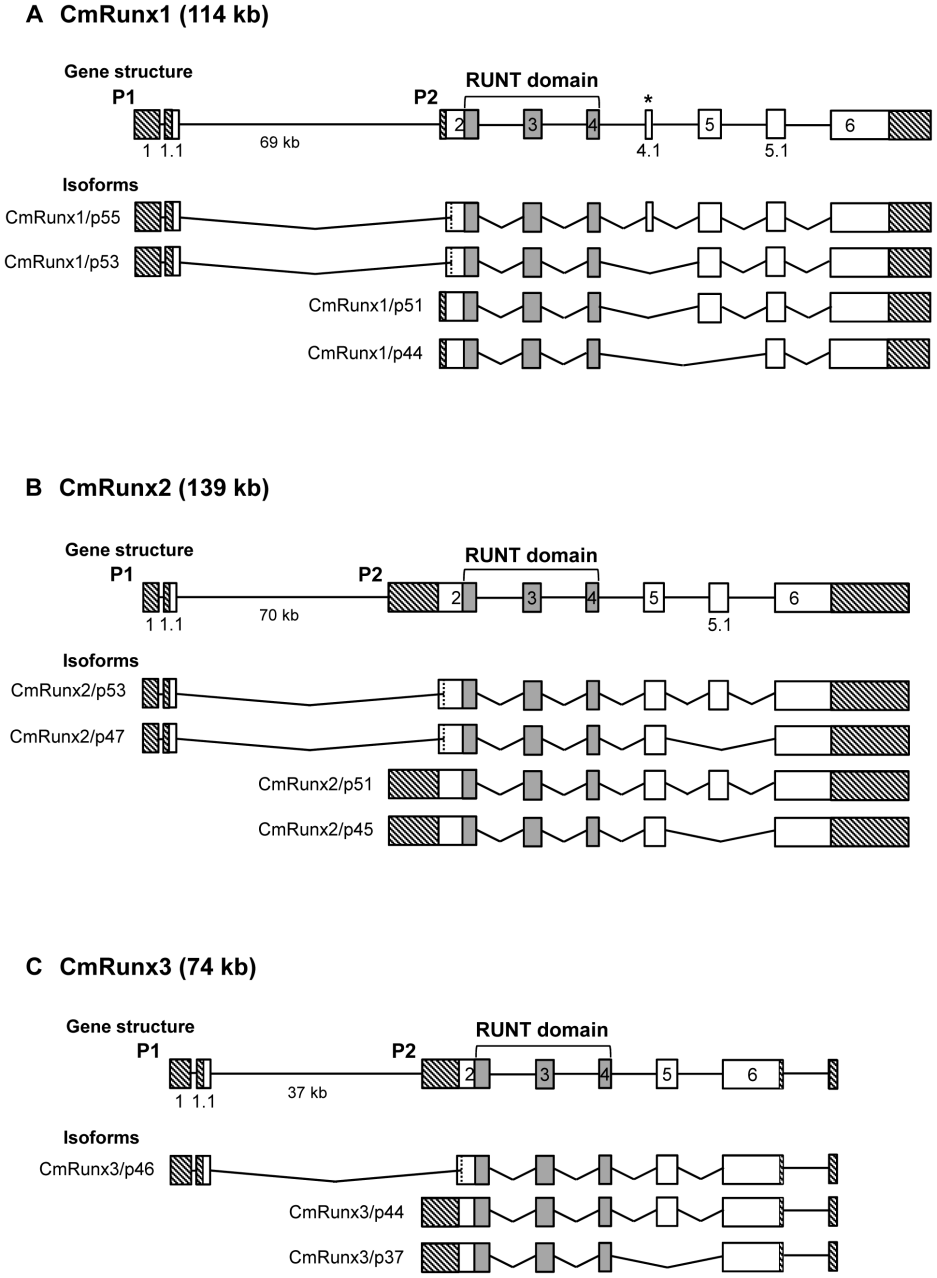
Exon-intron organization of elephant shark (*Cm*) *Runx* genes. Schematic representation of the gene structures and transcript isoforms of (A) *CmRunx1*, (B) *CmRunx2* and (C) *CmRunx3*. Exons are indicated by boxes. The vertical dashed lines indicate internal splice sites located within the coding exon. Exons constituting the Runt domain are indicated in grey. The two alternative promoters are denoted as P1 and P2. Crosshatched boxes indicate 5′- and 3′ UTRs. The asterisk (*) indicates an exon in *CmRunx1* that is absent in mammalian *Runx1*. Not drawn to scale.

**Figure 2:**
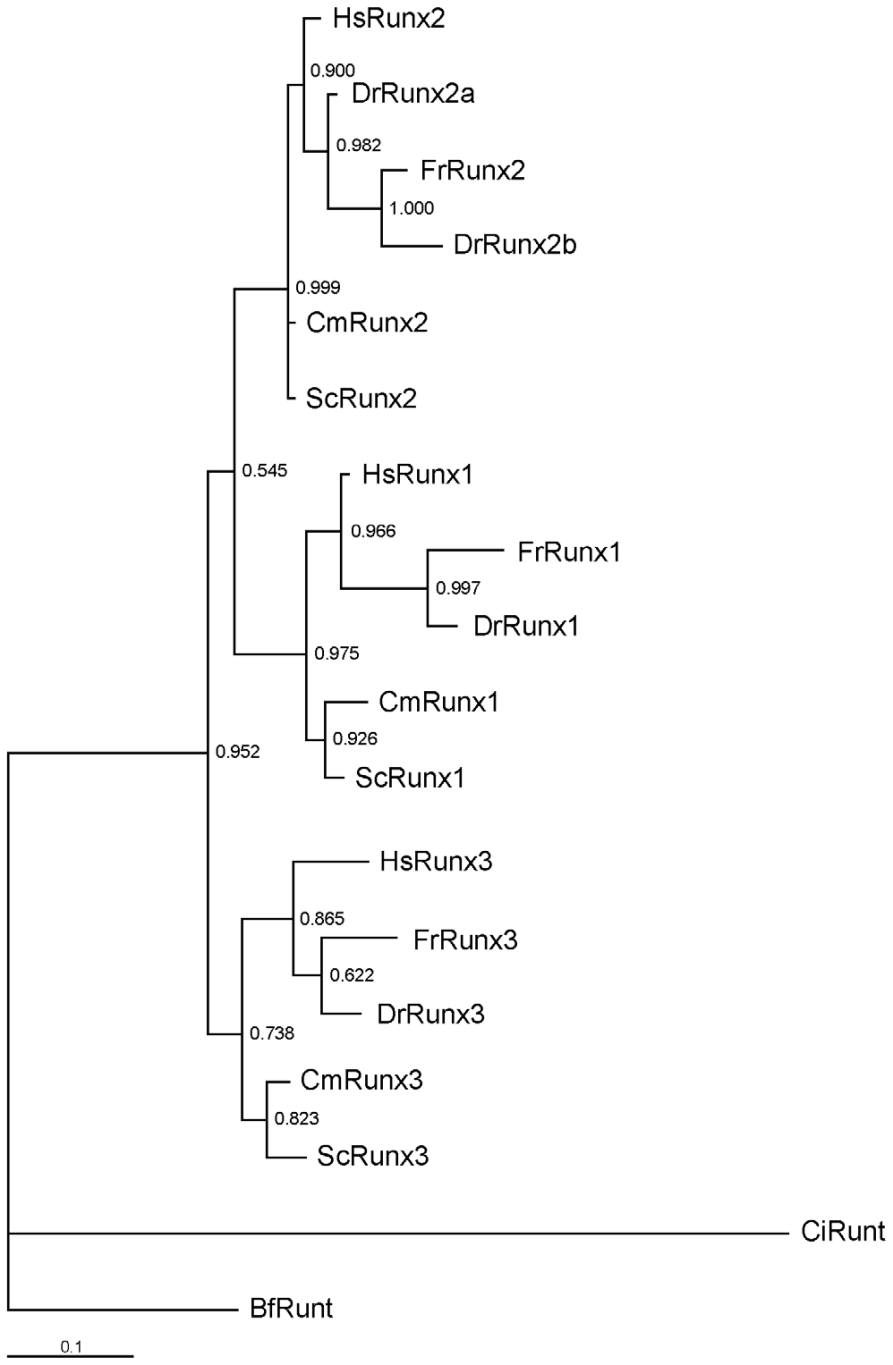
Phylogenetic tree (Bayesian inference) of chordate Runx sequences. Values adjacent to the nodes represent branch support (Bayesian posterior probability). Lancelet (*Branchiostoma floridae*) Runt (BfRunt) was used as the outgroup. Hs, human; Dr, *Danio rerio*; Fr, *Fugu* (*Takifugu*) *rubripes*; Cm, *Callorhinchus milii*; Sc, *Scyliorhinus canicula*; Ci, *Ciona intestinalis*.

To identify *Runx* genes in the elephant shark, we Blast-searched the elephant shark 1.4× coverage sequence assembly [31] using human RUNX protein sequences and identified scaffolds containing fragments of *Runx* genes. By designing primers complementary to selected exons of *Runx* and carrying out RT-PCR and RACE using gill and kidney cDNA as templates, we were able to obtain full-length coding sequences for three elephant shark Runx *α*-subunit encoding genes. These sequences were then mapped to the whole-genome assembly of the elephant shark [32], and their precise exon-intron boundaries, transcription start sites (TSS) and UTRs were determined (Fig. 1). To confirm the orthology of the three elephant shark genes, we carried out phylogenetic analysis together with Runx sequences from representative chordates. The phylogenetic analysis identified the three genes as *Runx1*, *Runx2* and *Runx3* (Fig. 2) that are located on scaffolds #152, #106 and #121, respectively.

All three elephant shark *Runx* genes encode a highly conserved Runt domain (Fig. 1). Additionally, like all mammalian *Runx* genes characterized to date, each of the elephant shark *Runx* genes contain two alternative promoters, P1 (distal) and P2 (proximal), that are separated by a characteristically large intron. The exon-intron boundaries of the three elephant shark *Runx* genes are largely conserved compared to their orthologs in human and other jawed vertebrates. However, *CmRunx1* was found to contain an extra exon (exon 4.1) (Fig. 1A) which has not been characterized in any of the known *Runx1* genes, except that of the dogfish, *S. canicula*, suggesting that this exon might be a chondrichthyes-specific feature. In addition, *CmRunx2* lacks the equivalents of exons 5.2 and 6.1 found in the mammalian *Runx2* gene [34] (Fig. 1B). These exons are also absent in chicken, frog and teleost fishes [26], [35], indicating that they were recruited in a mammalian ancestor and likely to perform functions that are specific to mammals. Among the three elephant shark *Runx* genes, *Runx3* is the shortest (*CmRunx1*, 114 kb; *CmRunx2*, 139 kb; *CmRunx3*, 74 kb) similar to its orthologs in mammals (*HsRunx1*, 261 kb; *HsRunx2*, 223 kb; *HsRunx3*, 65 kb; *MmRunx1*, 225 kb; M*mRunx2*, 211 kb; *MmRunx3*, 57 kb). The short sequence of the *Runx3* genes is likely due to the loss of an exon equivalent to exon 5.1 in *Runx1* and *Runx2* genes (Fig. 1).

Mammals express a variety of isoforms for each of the *Runx* genes arising from transcription initiation at two alternative promoters (P1 and P2) and alternative splicing of exons. Isoforms generated from the P1 promoter possess unique N-terminal sequences arising from transcription initiation in exon 1 and splicing that bypasses the P2 initiator ATG on exon 2. The use of alternative promoters contributes to the transcriptional and functional complexity of the *Runx* genes, as evidenced by expression studies and analyses of P1 and P2 promoter knockout mice wherein these transcripts display distinct patterns of expression and exert divergent biological functions during development [36], [37]. We could identify 2 to 4 isoforms for each of the elephant shark *Runx* genes that are homologous to the isoforms in mammals, indicating that the genomic organization and transcriptional profile of *Runx* genes were already complex in the common ancestor of jawed vertebrates.

**Figure 3:**
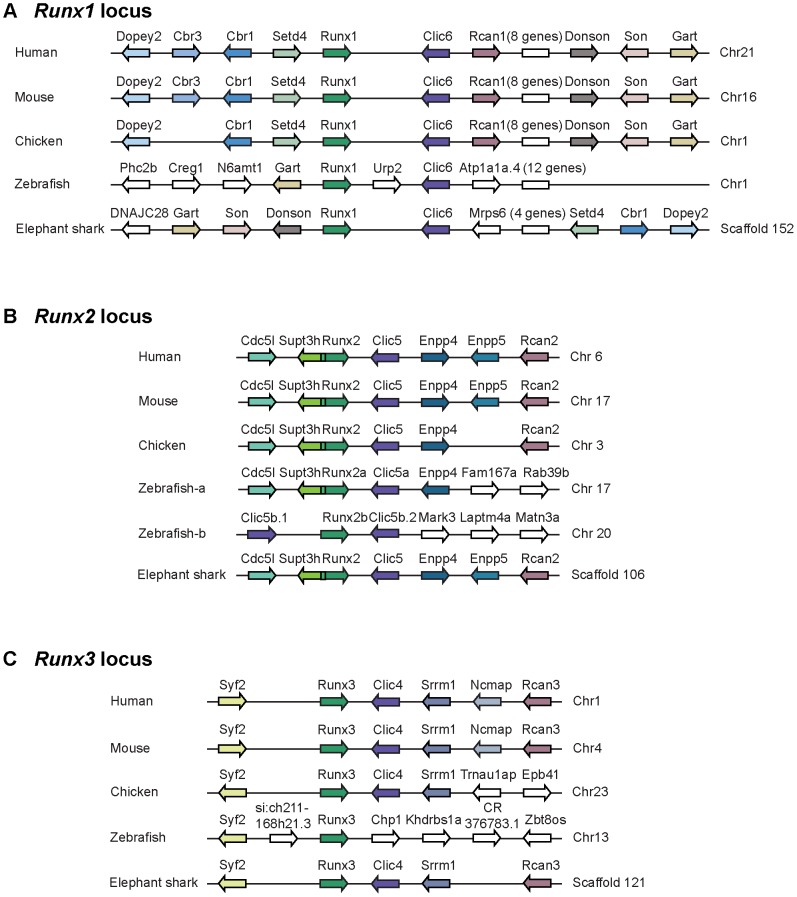
Synteny of genes in the *Runx* loci of elephant shark and selected bony vertebrates. (A) *Runx1* locus, (B) *Runx2* locus and (C) *Runx3* locus. Genes are represented by arrows. Genes with conserved synteny are coloured. Clusters of some non-syntenic genes are represented as white boxes and labelled in brackets. The gene order is from Ensembl (www.ensembl.org) and the elephant shark genome assembly (http://esharkgenome.imcb.a-star.edu.sg/).

The availability of the whole-genome sequence of elephant shark enabled us to compare the synteny of genes at *Runx* loci in elephant shark and other sequenced vertebrate genomes. The synteny of genes at the elephant shark *Runx1, Runx2* and *Runx3* loci is highly conserved in tetrapods (Fig. 3). A comparison of syntenic genes across the three *Runx* gene loci indicates that paralogs of *Clic* and *Rcan* genes are present in all three *Runx* gene loci. This indicates that three *Runx* gene loci in the jawed vertebrates are the result of duplication of an ancestral locus that comprised *Runx*, *Clic* and *Rcan* genes in that order. Of note are the characteristic “interlocked” gene structures of *Runx2* and *Supt3h* in all the jawed vertebrates, except the duplicated locus in zebrafish (*Runx2b* locus) in which *Supt3h* has been lost. Indeed, a *Supt3h* gene resides next to *Runt* gene in the genome of the most basally branching chordate, the amphioxus (chrUn: 270,089,714–270,297,951; Mar 2006-JGI1.0/braFlo1) indicating that their linkage is an ancient feature. It can therefore be inferred that linkage of *Supt3h* to *Runt* gene was retained in the jawed vertebrate *Runx2* locus whereas the paralogs of *Supt3h* in *Runx1* and *Runx3* loci were lost secondarily.

In contrast to the relatively larger blocks of conserved syntenic genes in elephant shark and tetrapod *Runx* loci, the conserved syntenic blocks in zebrafish *Runx* loci are shorter (Fig. 3). This indicates that *Runx* loci in zebrafish have experienced a higher degree of rearrangements, and that the arrangement of genes in human *Runx* loci is more similar to that of elephant shark than zebrafish, underscoring the importance of elephant shark as a useful model reference genome for studying the origin and evolution of human gene loci.

### Comparison of elephant shark Runx α-subunit protein sequences

**Figure 4:**
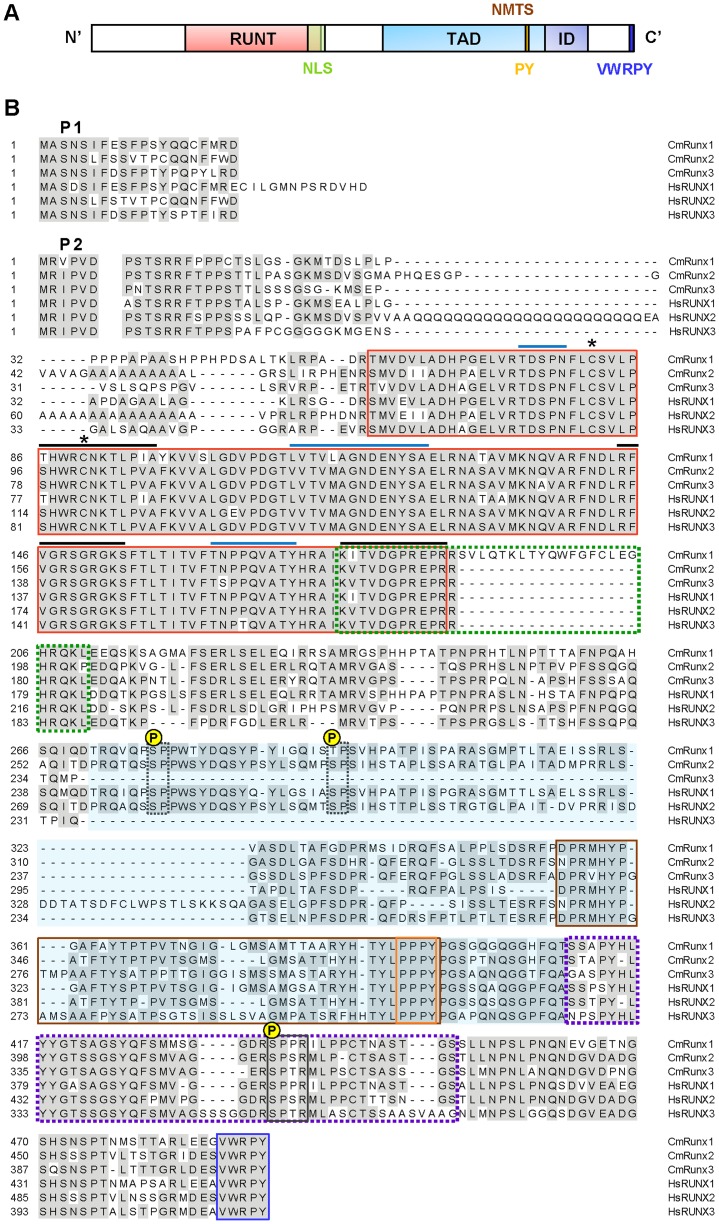
Runx α-subunit proteins Runx1, Runx2 and Runx3 in elephant shark and human. (A) Schematic representation of Runx α-subunit proteins. The characteristic domains found in all Runx proteins are indicated to show their relative positions along the protein. (B) Alignment of elephant shark and human Runx α-subunit proteins. The first block shows the amino-terminal part of the protein derived from the P1 promoter that differs from that derived from the P2 promoter. The highly conserved Runt domain is boxed with a red line. Within the Runt domain, surfaces involved in DNA contact and interaction with the β-subunit are denoted by black and blue lines, respectively. Cysteine residues involved in the redox regulation of DNA-binding activity are indicated with asterisks. Nuclear localization signal (NLS) is demarcated by a green dashed box. The PY and VWRPY motifs are indicated by orange and blue boxes, respectively. The transactivation domain (TAD) is highlighted in blue and the inhibitory domain (ID) is boxed by purple dotted lines. The nuclear matrix translocalization signal (NMTS) is boxed with a brown line. Minimal consensus sequences for phosphorylation by Erk or cdc2 are boxed by dashed and solid black lines, respectively. The residue targeted for phosophorylation is indicated by ℗. Cm, *Callorhinchus milii*; Hs, *Homo sapiens.*

Alignment of elephant shark Runx1, 2 and 3 protein sequences with human RUNX1, 2 and 3 sequences revealed several highly conserved protein domains. Among these is the 128 amino acid Runt domain which is almost totally conserved across elephant shark and human Runx proteins (Fig. 4). Within this domain, amino acid residues for DNA-binding as well as sequence motifs that interact with the β-subunit [38], [39] are well conserved. In addition, the nuclear localization signal (NLS), PY and VWRPY motifs, and sites of phosphorylation by Erk2/cdc2 (Fig. 4B) are also highly conserved. The PY and VWRPY motifs mediate transcriptional activity of Runx proteins by recruiting different interaction proteins. The PY motif in the transactivation domain mediates the binding of Runx proteins to WW domain-containing proteins, such as YAP and TAZ [40], [41]. Found invariably in all known vertebrate Runx proteins, the C-terminal pentapeptide VWRPY, is responsible for the repressive function of Runx proteins, through the recruitment of transcriptional co-repressors TLE/Groucho [42]. In human RUNX1, the serine residues S249 and S273 are each followed by a proline residue and acts as phosphorylation sites for ERK [43], [44]. These phosphorylation sites are also conserved in human RUNX2 (S280 and T305) and elephant shark Runx1 and Runx2. Additionally, the consensus phosphorylation site for CDC2, (S/T)PX(R/K), at which serine residue S451 of human RUNX2 was reported to be phosphorylated [45], is remarkably conserved in all human and elephant shark Runx proteins (Fig. 4B).

The N-termini of the P1 and P2 isoforms beginning with MAS(N/D)S and MR(I/V)PV, respectively, are highly conserved across all three Runx proteins between elephant shark and their mammalian orthologs. A distinctive feature present only in CmRunx1 (and its dogfish ortholog) is a short stretch of 18 amino acids downstream of the Runt domain (Fig. 4). This unique sequence is encoded by an extra exon 4.1 that is alternatively spliced in one of the isoforms of *CmRunx1* (Fig. 1). Whether these additional amino acids alter the structure and function of the CmRunx1 isoform is unknown, although one might speculate that the presence of this stretch within the nuclear localization signal (Fig. 4B, boxed in green dotted lines) might affect the isoform's nuclear translocation.

Of the three mammalian *Runx* genes, unique to *Runx2* is the Q/A stretch, an activation domain located N-terminal to the Runt domain that is composed of successive polyglutamine and polyalanine amino acids. Whilst human RUNX2 contains a stretch of 23 glutamine residues followed by 17 alanine residues, no glutamine repeats but a stretch of 10 alanine residues are found in elephant shark Runx2 (Fig. 4). The QA domain has been proposed to contribute to the osteoblast-specific function of Runx2. This domain controls transactivation function of the Runx2 and also inhibits the heterodimerization of Runx2 with the β-subunit [46]. In carnivoran mammals, the ratio of the glutamines to alanines (QA ratio) in Runx2 is strongly correlated with facial length [47]. In human, insertion in the alanine tract (23Q/27A) was observed in a CCD patient, suggesting that QA variations of RUNX2 may influence skeletal phenotype [48]. Indeed, glutamine repeat variants in RUNX2 have been recently found to be associated with a lower bone mineral density in the general human population [49]. Therefore, given the significance of the QA domain for skeletal functions of Runx2, it would be of interest to study the physiological relevance of this domain of Runx2 in cartilaginous fishes like the elephant shark which lack ossified endoskeleton.

Altogether, our comparisons indicate that protein domains of elephant shark Runx proteins are well conserved with those of human and demonstrate the highly conserved nature of Runx proteins in all jawed vertebrates.

### Expression profile of elephant shark α-subunit Runx genes

**Figure 5:**
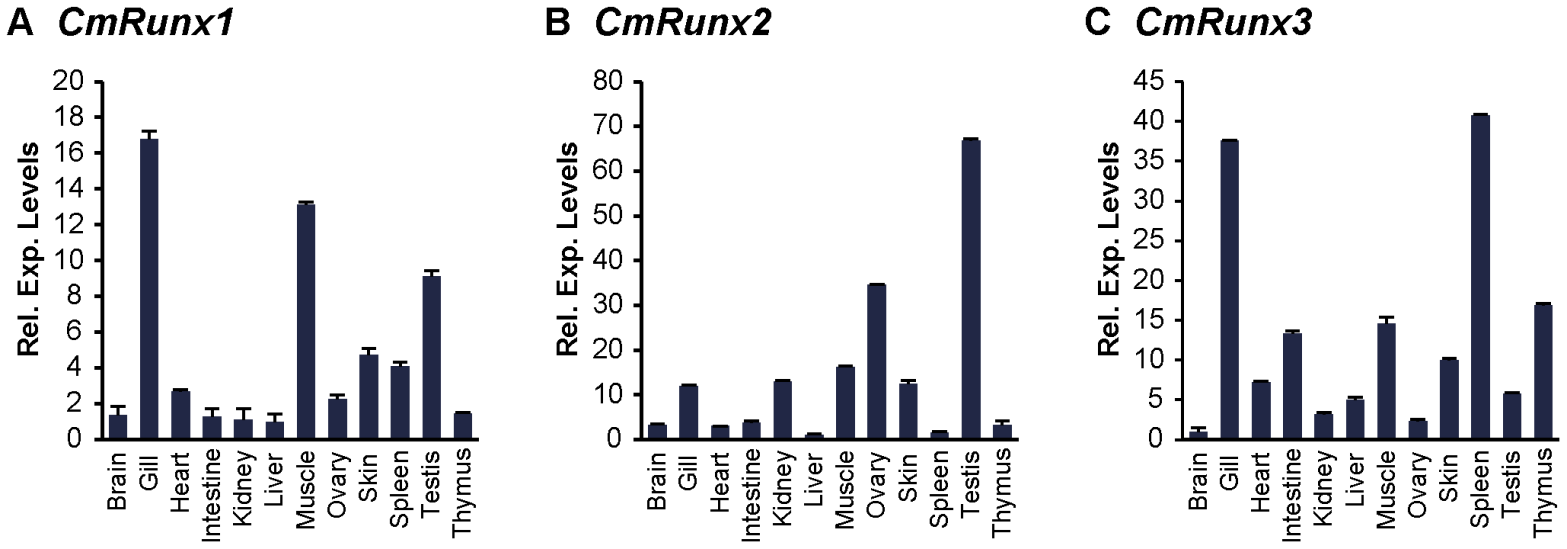
Expression patterns of elephant shark *Runx* genes. Relative expression levels of (A) *CmRunx1*, (B) *CmRunx2* and (C) *CmRunx3* in various tissues of the elephant shark determined by qRT-PCR.

We investigated the expression patterns of *Runx* genes in various tissues of adult elephant shark by quantitative RT-PCR. *CmRunx1* is highly expressed in the gills, muscle, testis, skin and spleen (Fig. 5A). High *CmRunx1* expression in the gill, a tissue enriched in blood cells, as well as the spleen, a lymphomyeloid tissue of the shark, is concordant with the integral function of Runx1 in hematopoiesis. In addition, high expression of *CmRunx1* in the muscle reflects its known role in mammalian skeletal muscle development. Although *Runx1* is not known to be expressed at high levels in testis of mammals, high expression of *Runx1* has been reported in another cartilaginous fish, the dogfish. Thus, Runx1 might have a function in testis that is specific to cartilaginous fishes. However, this hypothesis remains to be verified. Furthermore, a significant level of *Runx1* expression in the skin of the elephant shark is consistent with previous reports of *Runx* expression in placoid scales which are small conical structures in the skin of cartilaginous fishes [29].

Particularly striking is the significant expression of *Runx2* in the gonads (ovary and testis) of the elephant shark (Fig. 5B). This expression pattern is reminiscent of that in mice, in which ovary and testis were reported to be the predominant sites of non-skeletal tissue *Runx2* expression [50]. In human, recent investigations into the role of *RUNX2* in the ovary have highlighted its importance in ovulation and luteinisation [51], [52]. These congruent patterns of expression may suggest a role for *Runx2* in similar reproductive processes in the elephant shark. Given the indispensable role of *Runx2* in osteogenesis, analysis of *Runx2* expression in the cartilaginous/skeletal elements of the elephant shark would have been informative, but due to the unavailability of cartilage tissue from elephant shark, we could not verify expression of *Runx* genes in the cartilage. However, in the dogfish, all three *Runx* genes were found highly expressed in the visceral cartilage [29], indicating the possible prominent role of *Runx* genes in the unossified skeleton of cartilaginous fishes.

The immune system of cartilaginous fishes share several features common to other jawed vertebrates. These include tissue sites for immune cell production (thymus and spleen), specialized cell types for innate and adaptive immunity and genes encoding proteins for immune function, such as various immunoglobulin subtypes, TCR, MHC and cytokine-like molecules [53], [54]. The elephant shark *Runx3* is highly expressed in the gill, spleen and thymus (Fig. 5C), which are all considered to be lymphomyeloid tissues in cartilaginous fishes. Based on the well-established roles of *Runx3* in the development and function of diverse immune related cell types in mammals, it appears that *Runx3* may have already established roles in the development of the cellular immune system in the common ancestor of jawed vertebrates.

### Analysis of elephant shark α-subunit Runx promoter regions

**Figure 6:**
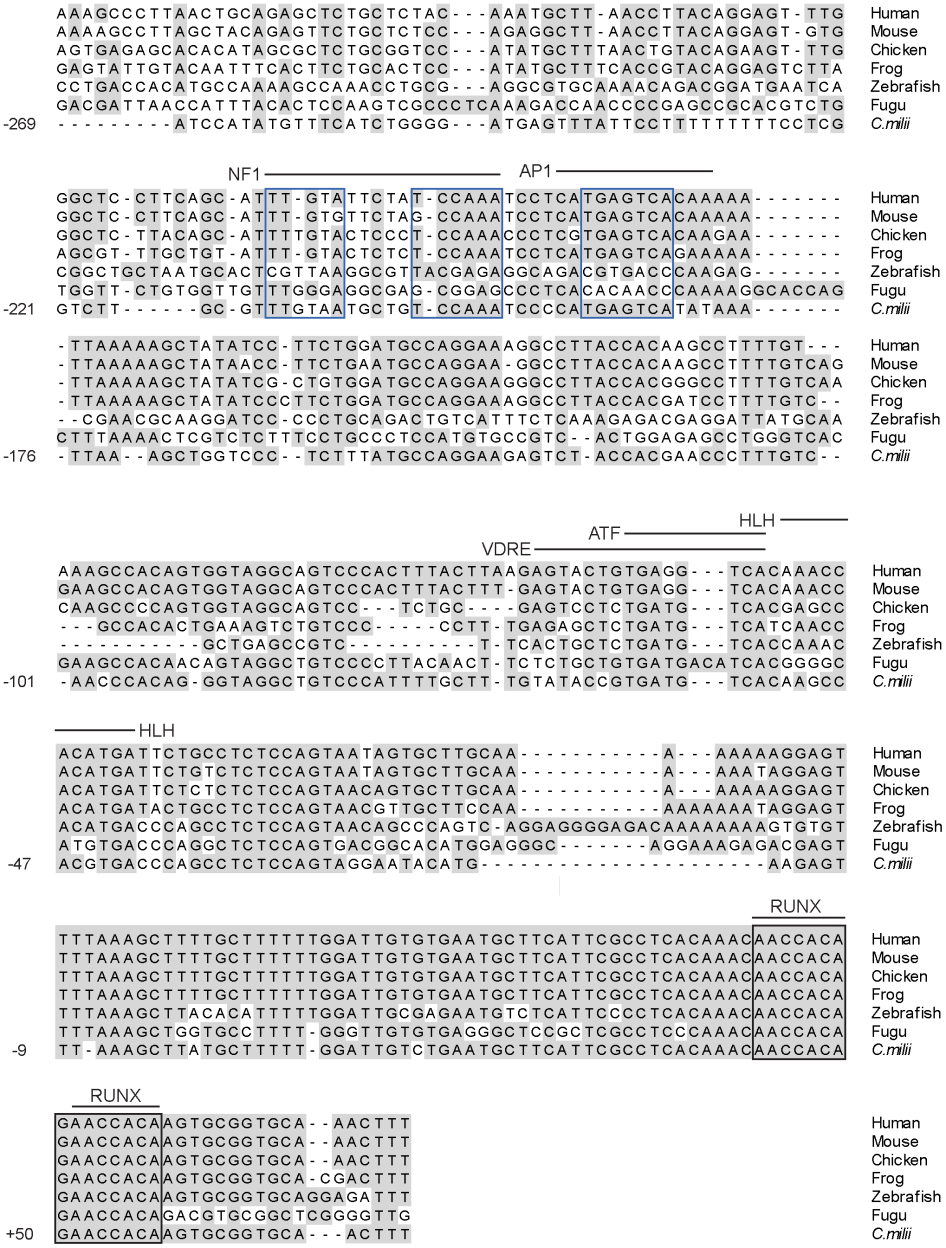
P1 promoter region of *Runx2* gene from elephant shark and selected bony vertebrates. Alignment of the P1 promoter regions is shown. The numbers to the left of the alignment indicate positions relative to the TSS (+1) of elephant shark. Sequences between –124 and -102 that contain insertions/deletions in some species are not shown. The conserved tandem Runx binding sites are boxed by black lines. Putative transcription factor binding sites are indicated by black solid lines and labelled. Core sites for NF1 and AP1 binding are boxed by blue lines.

The P1 promoter regions of mammalian *Runx1*, *2* and *3* genes harbour binding sites for transcription factors that are critical for their transcriptional regulation. In particular, P1 promoters of mammalian *Runx1, 2* and *3* contain two tandem binding sites for Runx. These sites have been implicated in auto and cross regulation of the three *Runx* genes [55], [56]. To verify if these binding sites are conserved in elephant shark, we compared the P1 promoter regions of *Runx1-3* in elephant shark, fugu/zebrafish and tetrapods. Remarkably, the tandem pair of Runx binding sites is totally conserved in P1 promoter regions of elephant shark *Runx1* (Fig. S1A), *Runx2* (Fig. 6) and *Runx3* (Fig. S1B) loci. The conservation of these binding sites in elephant shark and tetrapods indicate that the auto/cross regulation of the three *Runx* genes is an ancient feature of *Runx* genes and was present in the single ancestral *Runx* locus that gave rise to the three *Runx* loci in jawed vertebrates. Interestingly, in contrast to total conservation of these binding sites in elephant shark, they are less conserved in *Runx1* and *3* loci of fugu and zebrafish (Fig. S1 and 6). It remains to be seen if the divergent binding sites in teleosts are still functional and whether *Runx* genes in teleosts are subject to auto/cross regulation similar to that of elephant shark and tetrapods.

In addition to the two Runx binding sites, the P1 promoter of the mammalian *Runx2* locus contains well-characterized binding sites for other transcription factors that regulate the expression of *Runx2*. These include (i) a vitamin D response element (VDRE) with an overlapping ATF motif, that binds the VDR/RXR heterodimer and mediates the suppression of *RUNX2* transcription by the steroid hormone 1,25-(OH)2-vitamin D3 [57]; (ii) a Helix-loop-Helix consensus motif that is critical for the basal transcription of *Runx2*; and (iii) NF1 and AP1 motifs required for osteoblast-specific activity of the *Runx2* P1 promoter [58] (Fig. 6). All these binding sites, except the VDRE site, are conserved highly in the elephant shark *Runx2* gene, but to a lesser extent in the zebrafish and fugu *Runx2* genes (Fig. 6).

### Conserved noncoding elements in human and elephant shark α-subunit Runx loci

*Runx* genes exhibit highly specific expression in several tissues and cell lineages that is critical for their diverse roles in development and disease. However, the transcriptional regulation of *Runx* genes has not been well characterized. Comparative genomics is a powerful strategy for identifying evolutionarily conserved *cis*-regulatory elements. Since functional elements evolve slowly compared to their flanking sequences, *cis*-regulatory elements can be identified as conserved noncoding elements (CNEs) in the genomes of related species. Functional assay of CNEs in transgenic systems has indicated that many of them drive reporter gene expression in specific tissues [59]. Indeed, one such CNE predicted in the first intron of *Runx1* and conserved in human, mouse, rat, dog, horse and opossum, was shown to drive highly-specific expression to hematopoietic stem cells (HSCs) of mouse [60], indicating that it is an HSC enhancer.

**Figure 7:**
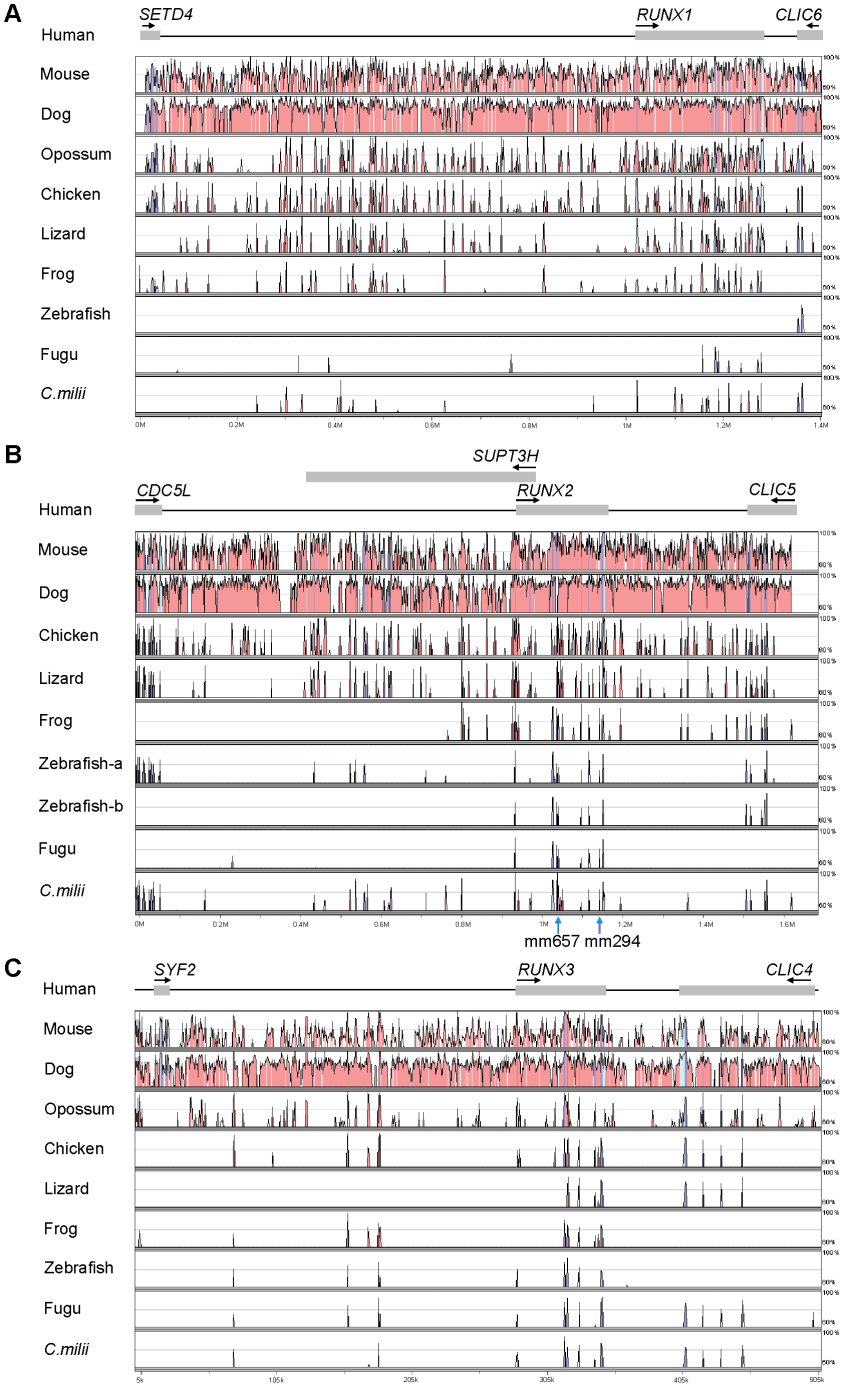
Conserved noncoding elements (CNEs) in *Runx* loci. VISTA plots obtained from the global alignment between the human, mouse, dog, opossum, chicken, lizard, frog, zebrafish, fugu and elephant shark (A) *Runx1*, (B) *Runx2* and (C) *Runx3* loci are shown. Sequence for opossum *Runx2* locus is not available. Human sequence is used as the reference sequence. Conserved sequences were predicted at a cut-off of ≥65% identity across >50 bp windows and are represented by peaks. Blue peaks denote conserved coding exons, pink peaks conserved noncoding regions (CNRs) and cyan peaks untranslated regions (UTRs). Experimentally verified CNEs (mm657 and mm924) in the *Runx2* locus are indicated with blue arrows below the x-axis and labelled.

The common ancestor of human and elephant shark diverged about 450 million years ago. Any CNEs conserved in these vertebrates over 900 million years of divergent evolution are likely to be functional elements and must be playing a fundamental role in the regulation of their associated genes. In order to identify such evolutionarily conserved *cis*-regulatory elements, we aligned the three human *Runx* loci with their corresponding orthologs from elephant shark and other jawed vertebrates and predicted CNEs that are >65% identical across 50 bp or more. Several CNEs that met these criteria were identified in the three *Runx* loci of human and elephant shark (Fig. 7) (Table S2). Notably, two of the CNEs located in the intronic regions of human *RUNX2* (Runx2_CNE7 and Runx2_CNE8) overlap two functionally characterized enhancers, denoted as mm657 and mm924 (Fig. 7B), that drive reporter gene expression in the branchial arches and facial mesenchyme respectively of transgenic mouse embryos [61]. The overlap of functional enhancers with the predicted human-elephant shark CNEs provides further support to the notion that CNEs conserved in human and elephant shark are likely to be functional *cis*-regulatory elements. Thus, they are strong candidates for functional assays that can help to identify enhancers of human *RUNX* genes.

Interestingly some of the CNEs conserved in the *Runx1-3* loci of human and elephant shark are missing in zebrafish and fugu (Fig. 7A and B), which is consistent with previous observations that CNEs in teleosts have been evolving faster than in elephant shark and other vertebrates [62]. This further emphasizes the importance of elephant shark as a model genome of choice for identifying evolutionarily conserved *cis*-regulatory elements in the human genome.

### miRNA binding sites in elephant shark α-subunit Runx genes

**Figure 8:**
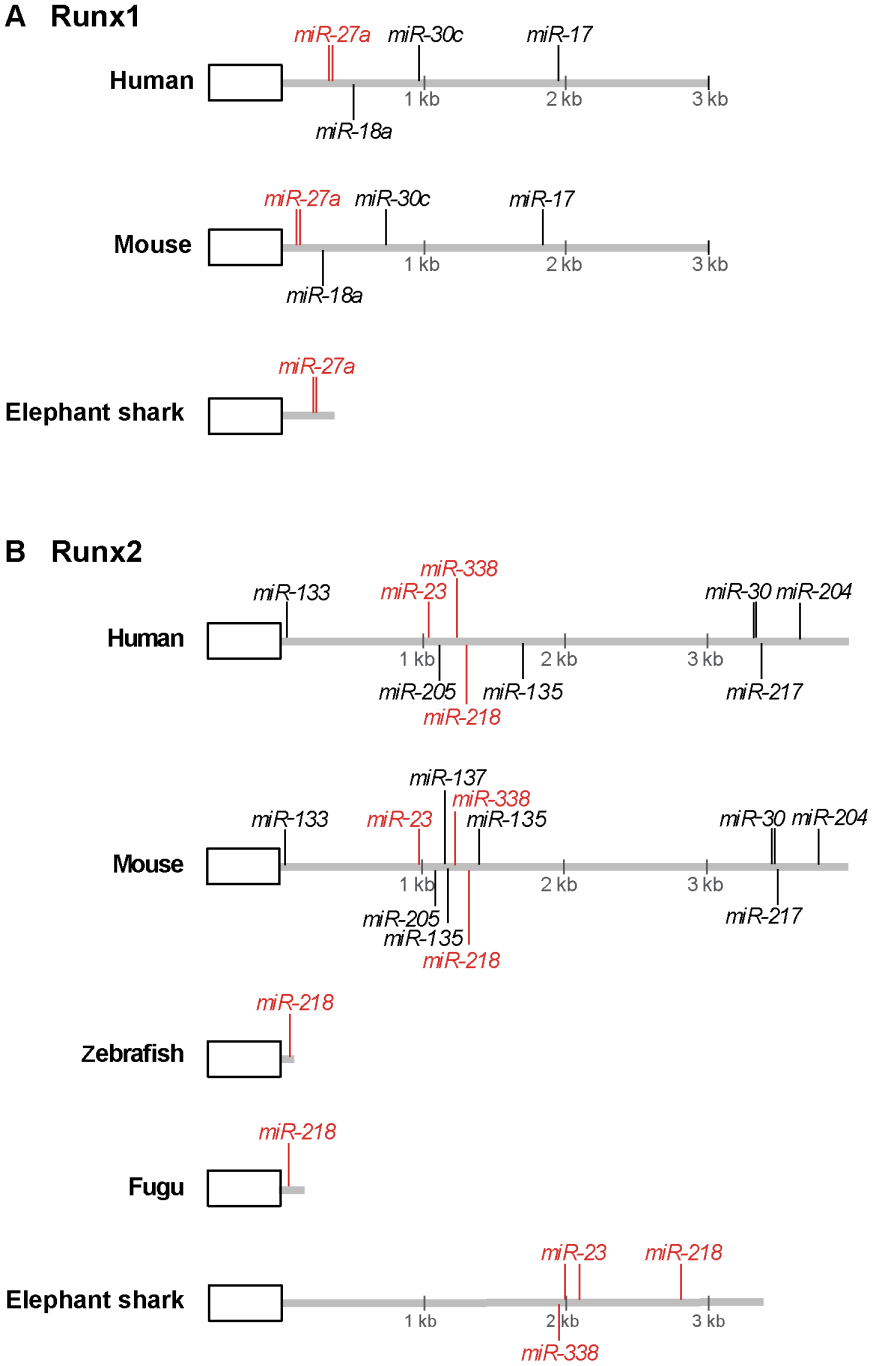
miRNA binding sites in the 3′UTR of *Runx1* and *Runx2* genes in elephant shark and selected gnathostomes. Schematic diagram of (A) *Runx1* and (B) *Runx2* 3′UTRs and miRNA binding sites. The last coding exon is represented by a rectangle and 3′UTR by a grey line. Positions of miRNA binding sites are indicated by vertical lines. Binding sites conserved in human and elephant shark are shown in red. Zebrafish and fugu *Runx1* loci are not shown as they do not contain any conserved miRNA binding sites.

Apart from transcriptional control by alternate promoters and *cis*-regulatory elements, expression of *Runx1-3* is also subjected to post-transcriptional regulation by miRNAs [63], [64], [65]. Several miRNAs have been shown to regulate *Runx1* expression levels and consequently its function in hematopoietic differentiation [64]. Among the highly conserved miRNA binding sites in mammalian *Runx1* 3′UTR, two clustered sites for miR-27a are remarkably well conserved in the 3′UTR of elephant shark *Runx1* but not in the teleost fishes, zebrafish and fugu (Fig. 8A). In mammals, miR-27 plays regulatory roles during megakaryocytic and granulocytic differentiation by attenuating *Runx1* expression and engaging in feedback loops with Runx1 [64]. Conservation of miR-27 binding sites, coupled with the presence of miR-27 in the elephant shark (GenBank Accession number JX994340) suggests that *CmRunx1* may similarly be post-transcriptionally controlled by miR-27 in hematopoietic lineages of the elephant shark.

Eleven Runx2-targeting miRNAs have been reported to control the physiological levels of Runx2 protein during osteogenesis and chondrocyte maturation [65]. Of these, binding sites for miR-23, miR-218 and miR-338 were found conserved in the 3′UTR of elephant shark *Runx2*, while only that for miR-28 is present in zebrafish and fugu *Runx2* (Fig. 8B). Since miR-23 (JX994633), miR-218 (JX994383) and miR-338 (JX994406) are present in the elephant shark, these conserved miRNAs are likely to be involved in the regulation of Runx2 expression in elephant shark, possibly during chondrogenic differentiation through mechanisms similar to that in other jawed vertebrates.

In contrast to *Runx1* and *Runx2*, no miRNA binding sites were found to be conserved in the rather short 3′UTR of elephant shark *Runx3* (data not shown).

### Cloning and characterization of elephant shark β-subunit Runx gene

**Figure 9:**
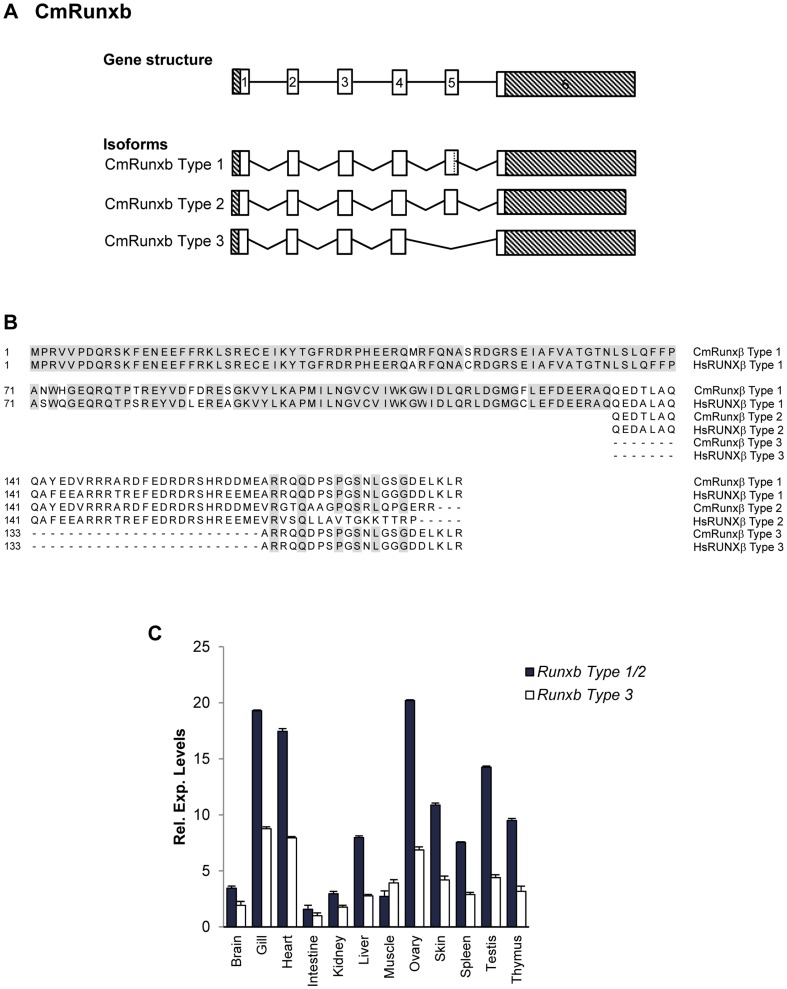
Exon-intron organization and protein sequence encoded by the elephant shark *Runxβ*. (A) Schematic representation of the genomic structure and the three transcripts cloned (*CmRunxb* types 1,2 and 3). Exons are indicated by boxes. The vertical dashed lines indicate internal splice sites located within a coding exon. The 5′ and 3′UTRs are represented as crosshatched boxes. (B) Alignment of elephant shark and human RUNXβ amino acid sequences using ClustalW. Conserved residues are shaded grey. Cm, *Callorhinchus milii*; Hs, *Homo sapiens*. (C) Expression patterns of elephant shark *Runxb* transcripts. Relative expression levels of *Runxb* Types 1+2 and Type 3 in various tissues of the elephant shark determined by qRT-PCR.

We cloned the full-length coding sequence of the elephant shark gene encoding the β-subunit of the CBF heterodimeric complex, *Runxb*. The exon-intron organization of elephant shark *Runxb* (*CmRunxb*) gene is identical to that of its human ortholog (Fig. 9A) [66]. Furthermore, *CmRunxb* is transcribed into at least three isoforms that are homologous to the human *RUNXB* type 1, 2 and 3 isoforms. The *CmRunxb* type 1, 2 and 3 isoforms encode proteins of 188, 185 and 156 amino acids, respectively (Fig. 9B). *CmRunxb* Type 1 and Type 3 isoforms are largely similar, except for the absence of exon 5 in the Type 3, as a result of exon skipping. *CmRunxb* Type 1 and Type 2 isoforms differ in their C-terminal ends, resulting from the use of alternative termination codon and splice donor/acceptor sites in exons 5 and 6 (Fig. 9A). At the protein level, CmRunxβ and human RUNXβ show high conservation of amino acid residues 1-165 (Fig. 9B), of which the N-terminal 135 amino acids required for its heterodimerization with the α-subunit and DNA binding [67] are almost perfectly conserved. We investigated the expression patterns of *Runxb* isoforms in various tissues of adult elephant shark by quantitative RT-PCR. All isoforms display a ubiquitous pattern of expression, with high levels of expression in the gill, heart, ovary and testis of the elephant shark (Fig. 9C). The functional significance of these isoforms that are conserved in human, elephant shark and other jawed vertebrates remains to be investigated. In an attempt to identify conserved *cis-*regulatory elements for *Runxb* locus, we searched for CNEs in the human and elephant shark *Runxb* loci but found none (data not shown).

## Materials and Methods

### Ethics statement

Elephant sharks were caught off the coast of Western Port Bay, Victoria (Australia) by licensed commercial fishermen. The samples used in this study were taken from animals that were already dead when the fishermen returned to the fishing jetty. Pieces of tissue were taken from the dead fish, frozen and transported to the laboratory for extraction of DNA and RNA [68], [69]. The extraction of DNA and RNA from frozen elephant shark samples was approved by the Institutional Animal Care and Use Committee (IACUC) of the Institute of Molecular and Cell Biology.

### Identification of Runx gene fragments in the elephant shark genome database

The elephant shark 1.4× coverage sequence assembly (http://esharkgenome.imcb.a-star.edu.sg/) was searched with human RUNX1, RUNX2, RUNX3 and RUNXβ protein sequences using ‘TBLASTN’ algorithm. The following contigs that contained fragments of various *Runx* genes were identified in the assembly: *Runx1*- AAVX01052077, AAVX01261782, AVX01381831 and AAVX01569464; *Runx2* - AAVX01175228, AAVX01545426, AAVX01631487, AAVX01048938, AAVX01307838 and AAVX01048937; *Runx3* - AAVX01039927, AAVX01288244, AAVX01157201 and AAVX01598430 and *Runxb*- AAVX01192354, AAVX01224391 and AAVX01471917.

### RT-PCR and 5′ and 3′ RACE

Primers were designed for representative exons of *Runx* genes identified in the elephant shark scaffolds and the full-length coding sequences of the genes were obtained by RT-PCR, 5′ RACE and/or 3′ RACE (primer sequences available upon request). 5′ and 3′ RACE were performed on single-stranded cDNA prepared from total RNA using the SMART RACE cDNA Amplification kit (Clontech, Palo Alto, CA) in a nested PCR. All RT-PCR and RACE products were cloned into the pGEM-T Easy Vector (Promega), and sequenced completely using the BigDye Terminator Cycle Sequencing Kit (Applied Biosystems, USA) on an ABI 3730xl capillary sequencer (Applied Biosystems, USA). During the course of this study, the whole-genome sequence of elephant shark became available [32]. The full-length cDNA sequences were mapped to the whole-genome assembly and the sequences of scaffolds that contained the genes (scaffolds #152, #106, #121 and #127) were extracted for further annotation and synteny analysis. Sequences for various isoforms of elephant shark *Runx* genes have been deposited in GenBank (Accession numbers KJ150227-KJ150240).

### Amino acid alignment and phylogenetic analysis

The protein sequences for human and other chordate *Runx* genes were retrieved from the National Center for Biotechnology Information (NCBI) database. Multiple sequence alignments were performed by using ClustalW. For phylogenetic analysis, gaps in the alignments were removed using Gblocks Server (ver. 0.91b) with default parameters [70]. The phylogenetic analysis was performed using MrBayes 3.2, employing Dayhoff+G as a substitution model and running 4 chains for 1,000,000 generations. Trees were sampled every 100 generations and according to a saturation curve of likelihood values, the first 2500 trees were discarded as burn-in. The *Runt* gene from *Branchiostoma floridae* was used as the outgroup.

### Expression profiling of elephant shark Runx genes by qRT-PCR

Total RNA was extracted from various tissues of adult elephant shark using Trizol reagent (Gibco BRL, Grand Island, NY) according to manufacturer's protocol. Purified total RNA was reverse-transcribed into cDNA with Superscript II (Invitrogen, Carlsbad, CA). The single strand cDNA was used as a template in qRT-PCR reactions with KAPA SYBR FAST qPCR Kit reagents (KAPA Biosystems, Boston, MA). Sequences of primers used in qRT-PCR are given in Table S1. All primer pairs span at least one intron which helps to distinguish cDNA from genomic DNA products. Expression levels of *Runx* genes were normalized using *β-actin* gene as the reference.

### Prediction of transcription factor binding sites (TFBS)

Analysis of TFBSs was carried out using JASPAR (http://jaspar.binf.ku.dk/cgi-bin/jaspar_db.pl). Only TFBS with prediction scores ≥ 9 were retained as putative TFBSs.

### Prediction of conserved noncoding elements (CNEs)

Genomic sequences of the *Runx* gene loci for the following species were extracted from Ensembl release 73 [71]: human (GRCh37 assembly, February 2009), mouse (GRCm38, December 2011), dog (CanFam3.1, September 2011), opossum (MonDom5, October 2006), chicken (GalGal4, November 2011), lizard (AnoCar2.0, May 2010), frog (JGI 4.2, November 2009), zebrafish (Zv9, July 2010) and fugu (FUGU5, October 2011). Repetitive sequences were masked using CENSOR at default settings [72]. Multiple alignments of *Runx* gene loci sequences were generated using the global alignment program MLAGAN (http://genome.lbl.gov/vista/index.shtml) with human as the reference sequence. CNEs were predicted using a cutoff of ≥65% identity across 50-bp windows and visualized using VISTA (http://genome.lbl.gov/vista/index.shtml).

## Supporting Information

Figure S1 P1 promoter region of *Runx1* and *Runx3* of elephant shark and selected bony vertebrates. Multiple sequence alignments of the P1 5′UTR regions of (a) *Runx1* and (b) *Runx3* are shown. The numbers to the left of the alignment indicate positions relative to the TSS (+1) of elephant shark (*C. milii*) genes. Corresponding regions from other bony vertebrates were aligned using ClustalW. The tandem Runx binding sites are boxed.
(PDF)

Table S1 Primers used for qRT-PCR.
(PDF)

Table S2 CNEs in the *Runx* loci of human and elephant shark.
(PDF)
