# Supplementary material for: Runx Family Genes in a Cartilaginous Fish, the Elephant Shark (Callorhinchus milii)
Source: PLoS One. 2014 Apr 3;9(4):e93816. doi: 10.1371/journal.pone.0093816 (PMC3974841; doi:10.1371/journal.pone.0093816)
Supplement: Figure S1 — P1 promoter region of Runx1 and Runx3 of elephant shark and selected bony vertebrates. Multiple sequence alignments of the P1 5′UTR regions of (a) Runx1 and (b) Runx3 are shown. The numbers to the left of the alignment indicate positions relative to the TSS (+1) of elephant shark (C. milii) genes. Corresponding regions from other bony vertebrates were aligned using ClustalW. The tandem Runx binding sites are boxed. (PDF) [file pone.0093816.s001.pdf]

Figure S1

A

```
      GTCACATGATTGGCA-AGAACCAATT----GAGATGGGCTGT------GGAAAGGGGAAC   Human
      GTCACGTGATTGGCA-AGAGCCAATG----GCGGTGGGCTGT------GGAAAGGGGAAC   Mouse
      GTCACATGGTTGGCA-AGAACCAATT----AAGATGGGCTGT------GGAAAGGGGAAC   Dog
      GTCACATGATTGGCA-AGCACCAATT----AAGATGGGCTGT------GGAAAGAGGAAC   Opossum
      GTCACATGATGGGTC-GGGGCCAATG----GAGTTGGGCTGT------GGAAAGGGGAAC   Platypus
      GTCACGTGACTCAAA-GGGACCAATG----GAGGCAGGTTAT------GGAAAGGGGAAC   Chicken
      GTCACGTGATGCAAA-CCAGCCAATA----AAGGGAGACTGCCAAAAAAAAAAGGGGAAC   Frog
+1    GCCACGTGATTACTGGAGCCCCAATT----GGATCGAGCTGC-------CAAAGGGGAAC   C.milii
      GACACACACACACAAAACACACACTTTTTAAAGCCATGCAGAATTCAAAGAAAGCAAACT   Fugu

      AGTTA-AATTTGT---------AATTTGGGTTGTGTGAAA-ACTTCTTTGGG-CCTCATA   Human
      AGTTA-AATTTGT---------AATTTGGGTTGTGTGAAA-ACTTCTTTGGA-CCTCATA   Mouse
      AGTTA-AATTTGT---------AATTTGGGTTGTGTGAAA-ACTTCTTTGGG-CCTCATA   Dog
      AGTTA-AATTTGT---------AATTTTGGTTGTGTGAAACACTTCTTTGGGGCCTCATA   Opossum
      AGTTA-AATTTGT---------AATTGGGGTTGTGTGAAACACTTCTTTGGGGCCTCATA   Platypus
      AGTTA-AATTTGT---------AATTTTGGTTGTGTGAAACACTTCTTTGGG-CCTCATA   Chicken
      AGCTACAATTTTTTTCTCAT-TATTTTGGGTTGTGTGCAAGACTTGTTTGGG-CCTCAGA   Frog
+50   AGTTA-ATTTTGT---------AATTTGGGTTGTGTGAAA---CTCTTTGGGCCCTCATA   C.milii
      TGCAAGATCGGGCAGACGACCTCTCTTTGGTGATGCTGAACACTGAGATTCATCCCTGCA   Fugu

         RUNX        RUNX
      AACAACCA----CAGAACC------ACAAGTTGGGTAGCCTGGCAGT---GTCAGAAGTC   Human
      AACAACCA----CAGAACC------ACAAGTTGGGTAGCCTGGCAGT---GTCAGAAGTG   Mouse
      AACAACCA----CAGAACC------ACAAGTTGGGTAGGCTGGCAGT---GTCAGAAGTC   Dog
      AACAACCA----CAGAACC------ACAAGTTGGGTACGGTGGCAGT---CACACAAGAC   Opossum
      AACAACCA----CAGAACC------ACAAGTTGGGTACCGTGGCAGT---CACAGAAGAG   Platypus
      AACAACCA----CAGAACC------ACAAGTTGGGTACAGAGGCAGT---CACACAAGAG   Chicken
      AGCAACCA----CAGAACC------ACAAGCTGCGC------GCAGA---CACTCGAGAG   Frog
+97   AACAACCA----CAGAACC------ACAAGTTGGGTGCAG-AACAGT---CACACAACAG   C.milii
      GACAAACAGTGGCAACAGGATTTAGCGAACCACAGACAAAAACAAGAGCCTAAAAAGCA   Fugu

      TGAACCCAGCATAGTGGTCAGCAGGCAGGA-CGAATCAC--------ACTGAATGCAAA-   Human
      TAAGCCCAGCACAGTGGTCAGCAGGCAGGA-CGAATCAC--------ACTGAATGCAAA-   Mouse
      TGCGCCCGGCATAGTGGTCAGCAAGCAGGA-CGAACCAC--------ACTGACTGCAAA-   Dog
      TGGGTCCGGGATATTGGTCAGCAGGCAGGG-CAAATCAC--------ACTGTCTGCAAA-   Opossum
      TGGGTCAGGGATATCGGTCAGCGGGCAGGG-CAAATCAT--------ACTGAGTAGAAA-   Platypus
      CGGGTCCGGGATATTGGTCAGCAGGCAGGG-CAAATCGC--------TTTGACTGCAAA-   Chicken
      TGGATCCGGGATATTGGTCAGCAGAAAGGGGCGAATCATTTTCGTTGACTGAAAAAAAAA   Frog
+143  TGGGTCCGGGATATTGGTCAGCAAATAGCAACTCATTCTGATTTAAACCACAGAGTG--   C.milii
      ATGAAGGAAGATA-TGGTGAAGGGAGAGAAAAGAGCGGA--------ACCAGAAAAGAG-   Fugu

      CCACA-GGGTTTCGCAGCGTG   Human
      CCACA-GGCTTTCGCAGAGCG   Mouse
      CCACA-GGGTTTCGCAGAGTG   Dog
      CCACA-GAGTTTCGTAGAGCG   Opossum
      CCACA-GAGTTTCGCAGAGCG   Platypus
      CCACA-GAGTTTCGTAGAGTG   Chicken
      CCACAAGACTTTCAGACATTG   Frog
+201  CCAAG-CGGCTTCGTAGAGTG   C.milii
      -CACTGAGCAACTGAAAAGTA   Fugu
```

B

```
                                                           RUNX
      TCGCATCCTGTGAGCTGAGGTTGGGT-TGACACTGGGAAGGCCTGGTCCCTCAACCACAG   Human
      TTGCATCCTGTGATGTGAGATTGGGT-TGACACTGGGAAGGTCTGGTCCCTCAACCACAG   Mouse
      TTGCATCCTGTGAGCTGAGGTTGGGT-TGACACTGTGAAGGCCTGGTCCCTCAACCACAG   Dog
+1    CTTCGGGCTCAGAACACTGGTTGAGC-TGACACTCTGTCGGGCCTCACTTTGAACCACAG   C.milii
      TTCAAAGCTCCCTGCAAACCTCAGCCACAACAATTTAATGGGCTTCTGCACGGGTTTCAC   Fugu

       RUNX
      AACCACAAGGCCAG---GCCCTT-GCCGCCTCCAGGGCCCTGCGCGGGAGCT--GGTTGG   Human
      AACCACAAGGCCAG---GCCCTC-GCCGCCTCCAGGGCCCTGCG-AGGAGCT--GGTTGG   Mouse
      AACCACAAGGCCAG---GCCCTCAGCCGCCTTCAGGGCCCTGCGCGGGAGCT--GGTTGG   Dog
+60   AACCACAAGTGTGGTGTGGTGCAGCCTACTTCTCAGGACTTGCACCTGAGTCTCAGTGGG   C.milii
      C-CCAGAAGGAAGACGGAATGCTTCCAAAAGTTGTGG----GCAACAAAGTGAAAATTGA   Fugu

      CTCCTGGTGCTCCCCACCCCC-GGCCGCCCTCGTACCCACCAGAGCCTGGGCTCTGTCAA   Human
      TTCCTGGTG-TCCCCACCCCCTGGCCACCCTGGCCCCTGCCACAGCCTGGGCTCCGTGGA   Mouse
      CTCTTGGTCCTCCCCACCCCCGGGCCGCCCTCGCACCCACTGGAGCCCGGGCTCTGTTGG   Dog
+120  TTGCTAGATATTTTTTTTAAAAAAACAGGCT---ACCCACCACCAACTGGACCTCGGCAC   C.milii
      GAGCGAAGATGGAGGGTCAGGAGGAGAGATTCAGAGAAACAACGATCAAGTAAAGAGAAG   Fugu

      GGGTAA   Human
      GGGTGA   Mouse
      GGGTGA   Dog
+177  GCAGAG   C.milii
      GCAAAG   Fugu
```
