# Supplementary material for: Runx Family Genes in a Cartilaginous Fish, the Elephant Shark (Callorhinchus milii)
Source: PLoS One. 2014 Apr 3;9(4):e93816. doi: 10.1371/journal.pone.0093816 (PMC3974841; doi:10.1371/journal.pone.0093816)
Supplement: Table S1 — Primers used for qRT-PCR. (PDF) [file pone.0093816.s002.pdf]

Table S1. Primers used for qRT-PCR.

Gene	Primer Name	Primer Sequence (5'→ 3')
CmRunx1	CmRUNX1 FW	CAACAAAACCCTCCCCATCGCCTA
	CmRUNX1 RV	CTGTGATTTTGATGGCTCTGTGG
CmRunx2	CmRUNX2 FW	AGCTATAAAGGTTACAGTGGATGGA
	CmRUNX2 RV	CTGGCCTTGAGAACTGAAAGGAA
CmRunx3	CmRUNX3 FW	TGCTGAGTAGTTTTCATCGTTTCC
	CmRUNX3 RV	AGATGAGCGAGCCGGTGTC
CmRunxb Type 1/2	CmRUNXB T1/2 FW	CACCAACCCGAGAGTATGTGGAC
	CmRUNXB T1/2 RV	TCCTCATATGCCTGCTGAGCTAAG
CmRunxb Type 3	CmRUNXB T3 FW	ATCGAGATGACATGGAGGCACGCA
	CmRUNXB T3 RV	CTAGCGTAGTTTGAGTTCATCTCC
CmActin	CmActin FW	GGTATTGTCACCAACTGGGA
	CmActin RV	AGATGGGCACAGTGTGGGTG
