# Supplementary material for: Runx Family Genes in a Cartilaginous Fish, the Elephant Shark (Callorhinchus milii)
Source: PLoS One. 2014 Apr 3;9(4):e93816. doi: 10.1371/journal.pone.0093816 (PMC3974841; doi:10.1371/journal.pone.0093816)
Supplement: Table S2 — CNEs in the Runx loci of human and elephant shark. (PDF) [file pone.0093816.s003.pdf]

Table S2. CNEs in the *Runx* loci of human and elephant shark.

CNE ID	Human *Runx* CNE coordinates (hg19)	Length (bp)	% identity	Location
Runx1_CNE1	Chr21:37204095-37204220	126	73	*Setd4-Runx1* intergenic
Runx1_CNE2	Chr21:37143334-37143451	118	76	*Setd4-Runx1* intergenic
Runx1_CNE3	Chr21:37031066-37031297	232	76	*Setd4-Runx1* intergenic
Runx1_CNE4	Chr21:36341746-36341933	188	72	*Runx1* intron
Runx1_CNE5	Chr21:36190839-36190995	157	76	*Runx1* intron
Runx2_CNE1	Chr6:44526642-44526813	172	74	*Cdc5l-Supt3h* intergenic
Runx2_CNE2	Chr6:44886521-44886644	124	71	*Supt3h* intron
Runx2_CNE3	Chr6:44900237-44900389	153	77	*Supt3h* intron
Runx2_CNE4	Chr6:44988709-44988911	203	71	*Supt3h* intron
Runx2_CNE5	Chr6:45164020-45164128	109	82	*Supt3h* intron
Runx2_CNE6	Chr6:45305281-45305382	102	78	*Runx2* intron
Runx2_CNE7	Chr6:45401543-45401611	69	90	*Runx2* intron
Runx2_CNE8	Chr6:45506422-45506519	98	74	*Runx2* intron
Runx3_CNE1	Chr1:25501275-25501393	119	73	*Syf2-Runx3* intergenic
Runx3_CNE2	Chr1:25394035-25394210	176	70	*Syf2-Runx3* intergenic
